# Jugglers and tightrope walkers: The challenge of delivering quality community pharmacy services

**DOI:** 10.1371/journal.pone.0200610

**Published:** 2018-07-23

**Authors:** Margaret C. Watson, Zoe C. Skea

**Affiliations:** 1 Department of Pharmacy and Pharmacology, University of Bath, Claverton Down, Bath, England; 2 Health Services Research Unit, University of Aberdeen, Foresterhill Campus, Aberdeen, United Kingdom; University of New South Wales, AUSTRALIA

## Abstract

**Introduction:**

This is the first exploration of service providers’ attitudes and beliefs of quality and quality improvement in the community pharmacy setting in the UK.

**Materials and methods:**

A series of interviews and focus groups was conducted with stakeholders from the pharmacy profession in the UK. Interviews were semi-structured and conducted face-to-face or by telephone. Focus groups were conducted with naturally-occurring groups i.e. at pharmacy conferences. Interviews and focus groups were audio-recorded, transcribed and analysed systematically using an interpretive approach.

**Results:**

Forty-two individuals participated across four focus groups and four interviews. A maximum variation sample was achieved in terms of pharmacist and pharmacy characteristics. Participants were generally positive about the need for quality and quality improvement and provided multifaceted and interlinked interpretations of quality and acknowledged its dynamic nature *“quality moves forward”*. The challenge of standardising practice whilst providing person-centred care emerged: “*you don’t want to lose the personal touch*, *but you can’t have people having a variable experience and one day it’s fantastic and the next day it isn’t”*. A variety of quality measurement methods were identified including direct observation (by internal and external agents) and feedback (mystery shoppers, colleagues, regulatory inspectors, service users), suggesting that standardisation was also needed in terms of future quality measurement. There was a tendency to report negative events as triggers for improvement. Future initiatives could adopt more positive approaches including positive deviants “*There’s nothing more powerful than people who’ve come up with something really good sharing it with their other colleagues*”.

**Discussion:**

The results are being used to develop and evaluate future quality improvement initiatives in this sector. These are likely to be targeted at organisational, team and individual levels.

## Introduction

Around 18 million general practice consultations and 650,000 emergency department (ED) consultations occur annually in the UK for conditions which could be treated effectively in community pharmacies[1], hereafter referred to as acute consultations. The Keogh report urged that ‘urgent but non-life threatening’ conditions be treated in non-secondary care settings and identified community pharmacies as an ‘under-utilised resource’ which could substantially reduce the burden on other healthcare providers[2]. Furthermore, equivalent health outcomes can be achieved for these conditions with care provided from community pharmacies[3] compared with ED- and general practice-provided care.

Demand for these consultations needs to be re-directed from high-cost settings to effective and cheaper alternatives, including community pharmacies and self-care. However, redirection needs to address accessibility, affordability, availability and most importantly, safety of care[4]. The public not only needs to be made aware of community pharmacy as an alternative source of healthcare, but also needs reassurance that the quality of care in this setting would be equivalent to that delivered by other healthcare providers. Evidence suggests, however, that quality improvement is needed for the management of these consultations in community pharmacies[5–7]. To date, there has been no exploration of the concept of quality regarding the management of acute consultations in this setting. Against this backdrop, this is the first exploration of service providers’ attitudes and beliefs of quality and quality improvement in the community pharmacy setting in the UK.

## Material and methods

### Study design

A series of focus groups and interviews was undertaken with the method used varying according to participant availability and preference.

### Recruitment, sampling and consent

Purposive, convenience and snowballing techniques[8] were employed to derive the sample. Our sample size was guided by the concept of data saturation and we anticipated that up to 40 participants would be sufficient to identify a range of experiences and views[9,10]. Key informants from pharmacy organisations were purposively sampled (based upon their role within an organisation or their involvement with quality improvement) identified through professional and personal networks and invited to participate by email. Convenience sampling included recruitment of groups of individuals from scheduled events. The Royal Pharmaceutical Society in Scotland (RPSiS) agreed that focus groups could be undertaken during the national conference in August 2015. Similarly, the Royal Pharmaceutical Society (RPS) United Kingdom (UK) agreed for a focus group to be held during the annual conference in September 2015. For both conferences, information about the focus groups was sent to participants in advance of the events. RPSiS conference participants were advised of the time and venue for the focus group and asked (by conference organisers) to register to participate. RPS (UK) delegates were asked to contact the researcher by email in advance if they wished to participate. Snowball sampling was undertaken with interviewees suggesting additional key informants who were then invited to participate. This included one interviewee who suggested conducting a focus group with a pre-existing group of superintendent pharmacists and acted as a conduit for access to this group.

### Data collection

One researcher (MW) conducted all the data collection except for one focus group (FG2) which was undertaken by a senior researcher from MW’s academic department. Both focus group facilitators were female, PhD-qualified researchers with over 20 years’ experience of health services research and both were qualified pharmacists. The focus groups/interviews were conducted face-to-face or by telephone (Table 1). The topic guide was informed by existing work on quality and quality improvement and the same guide was used for interviews/focus groups (S1 Appendix). All participants gave written consent before participating in interviews/focus groups.

**Table 1:** Characteristics of focus group and interview participants.

Activity/Duration (minutes)	Details	Participants	Characteristics
**Focus Group: 1 (FG1)** **Face-to-face (49)**	Delegates attending RPSiS National Conference.	17	Female (F):Male (M), 12:5 Owner (4); locum (4*); Pre-registration pharmacy students (2); other e.g. addiction/mental health/health board/general practice role (3); Unknown (4).
**Focus Group: 2 (FG2)** **Face-to-face (35)**	Delegates attending RPSiS National Conference.	13	F:M 9:4 Owner (2); employee (2); locum (1); Pre-registration pharmacy students (3); other e.g. addiction/mental health/health board/general practice/industry role (3); Unknown (2).
**Focus Group: 3 (FG3)** **Face-to-face (50)**	Delegates attending RPS (UK) Conference.	5	All female Locum (1); Employee pharmacist, medium-chain (1); Large chain, head office pharmacists (3)
**Focus Group: 4 (FG4)** **Telephone (49)**	Superintendent Pharmacists.	3	All female. All large chain superintendents.
**Interviews** **(ID1 to 4)** **Face-to-face (n = 1) (69). Telephone (n = 3) (30, 52, 55)**	Key stakeholders: pharmacy and/or public health	4	All male. ID1, Senior Executive Community Pharmacy Contractor organisation ID2, Senior Executive Community Pharmacy ID3, Community Pharmacy experience with specific quality improvement role ID4, Community pharmacy experience and specific quality improvement role

RPS(iS) Royal Pharmaceutical Society (in Scotland) CEO Chief Executive Officer F Female M Male

*Also represented national contractor organisation.

### Data analysis

Interviews and focus groups were audio-recorded and transcribed verbatim. Our approach to analysis was systematic and interpretive i.e. to explore how pharmacy personnel interpreted and made sense of their experiences[11]. The two authors familiarized themselves with the whole dataset and following initial familiarization with transcripts, developed a coding framework based on *a priori* questions and emergent themes. Initial codes (text labels) from this framework were systematically applied to the data. Data management and initial analytic coding was facilitated by the use of NVivo 10 text software. The primary focus during the analysis was on the *a priori* study aims. Particular attention was paid to: the types of judgement, beliefs and attitudes (including concerns) that participants expressed in relation to quality and quality improvement in the context of community pharmacies; and views relating to strategies for assessing and improving the nature of community pharmacy consultations. (Participants’ views regarding the role of patients and public in quality improvement in community pharmacy services was explored but will be presented elsewhere.) This study is reported in accordance with COREQ[12].

### Consent and ethical review

Ethical review and approval for the study was provided by the College Ethics Review Board, University of Aberdeen (CERB/2015/6/1208).

## Results

### Characteristics of the study subjects

In total, 42 service providers participated in the study. Four focus group discussions were undertaken with 38 pharmacists/pharmacy support staff and individual semi-structured interviews were undertaken with four key informants from pharmacy organisations across the UK (Table 1). The interviews and focus groups lasted an average of 45 and 46 minutes, respectively. For the individual interviews, 11 key informants were contacted, six responded and five agreed to participate. One interview could not be completed within the timescale of the study and one individual could not participate due to a potential conflict of interest with a new policy development. No reason was given for non-participation/non-response by the four remaining individuals (one of whom changed professional role shortly after the invitation was sent).

### What does quality mean to community pharmacy service providers?

Participants were asked about their understanding of the definition of quality in relation to community pharmacies in general and more specifically in relation to acute consultations. Although participants were positive about the need for quality and quality improvement in this setting, their descriptions of quality were multifaceted and interlinked, varying both within and across participants. There was acknowledegement that quality is not one single entity *“Quality is quite an abstract term”* (Int4); that it is dynamic *“quality moves forward”*(FG4) and what might be considered good quality currently may become obsolete as services develop: *“We’re in a changing landscape and it would be foolish to think we could just stay as we are” (FG4)*.

Participants identified the importance of measuring quality of care: *“If you don’t measure what you’re doing then nobody is going to be interested in getting you to do it…*.*we can only demonstrate …how many activities we’ve done; that’s not a measure of quality” (Int1)*. Quality was described in terms of: a) Relational aspects of care (showing empathy, developing rapport); b) instrumental aspects of care (eliciting specific information during consultations; providing the right information/advice; and c) ensuring good clinical outcomes for patients (prompt resolution of symptoms.) No single dimension was suggested as more important than another—in other words they valued all aspects. Participants emphasised the importance of, and their desire, to achieve positive patient outcomes including patient satisfaction:

*“you obviously want to please your customer and do the right thing by them but they don’t always* ..*know what the right thing is”*

*(FG4)*.

Several participants discussed their views about the need to improve quality and there was a general acceptance of a need for more standardisation/consistency of practice both within and across pharmacies. However, there was also recognition of the challenges inherent in any drive to standardise practice whilst also providing ‘person-centred’ individualised care. Whilst there was general agreement that standardisation or consistency was a valuable endeavour and potentially achievable (i.e. attempting to agree and achieve a minimum standard), there was also an expressed preference that any move towards standardisation should retain a degree of flexibility:

“*you don’t want to lose the personal touch*, *but you can’t have people having a variable experience and one day it’s fantastic and the next day it isn’t*!

*(FG4)*.

The need for continuity of care was also discussed in the context of a changing workforce:

*“you want consistency at whatever time of the day that that[sic] patient goes in*, *so you want the same level of service if someone goes in at 10 o’clock at night as they would get at midday.”*

*(FG4)*;

*“patients still say they don’t actually know who the pharmacist is*, *because of longer working hours we don’t necessarily have continuity of pharmacy staff”*

*(FG1)*.

Whilst there was general endorsement amongst participants of the need to deliver high quality services and become more actively engaged in quality improvement of community pharmacy services, there was also the suggestion that the appropriate management of acute consultations was not a priority for some pharmacists:

*“there is an element of pharmacists focusing on the dispensing side*, *the services side*, *and you are almost forgetting that the OTC* [over-the-counter]*-part is their accountability as well*, *the quality of conversation*, *the advice*, *the product recommendations sit under their remit”*

*(FG3)*.

The tension between commercial enterprise and healthcare provision was discussed with the management of acute consultations considered to be lacking in importance compared with other more lucrative processes i.e. dispensing:

*“we don’t place enough importance on it [managing acute consultations] because of ‘that number thing’ and therefore people feel that that’s the most important element* ..*in terms of patient contact it’s significant, more significant than it looks* ..*and I think it’s the shop window if you like*, *it’s the entry point for a lot of things and we’ve dumbed it down because we haven’t been focused on it”*

*(Int1)*.

Leadership of the profession, as well as within individual pharmacies, was highlighted as an important influence on quality and engagement with quality improvement. It was suggested that the profession’s leaders need to engage with quality improvement and to recognize that small changes across a large number of pharmacies would be beneficial. There was recognition that quality improvement could be undertaken on a small scale at a local level by pharmacists who could then become “*advocates” (Int 2)*:

*“I think leaders need to talk about quality improvement far more than we have done in the past*.*”*

*(Int2)*.

Quality improvement within pharmacies was considered to be underdeveloped and not yet embedded within the culture. It was noted, however, that the profession’s focus on safety could be regarded as quality improvement:

*“I think that the general culture of pharmacy is to* ..*achieve the standard and maintain the standard and that’s accurate dispensing or..customer satisfaction…using a standardised or formalized proven quality improvement technique is absolutely in its infancy in community pharmacy.”*

*(Int3)*.

### What quality measures are used in community pharmacy?

There was considerable variation regarding whether and how quality was measured, with the suggestion that a mismatch currently exists between the measurement of activity rather than quality or patient outcomes:

*“Dispensing is all predicated around the numbers and doing it efficiently and fast and giving the patient a good service from that point of view. It’s not necessarily focused on* ..*making sure that people get the best outcome from the medicines that are being dispensed. We don’t really measure anything that’s necessarily relevant to patient outcomes”*

(Int4).

**Fig 1:**
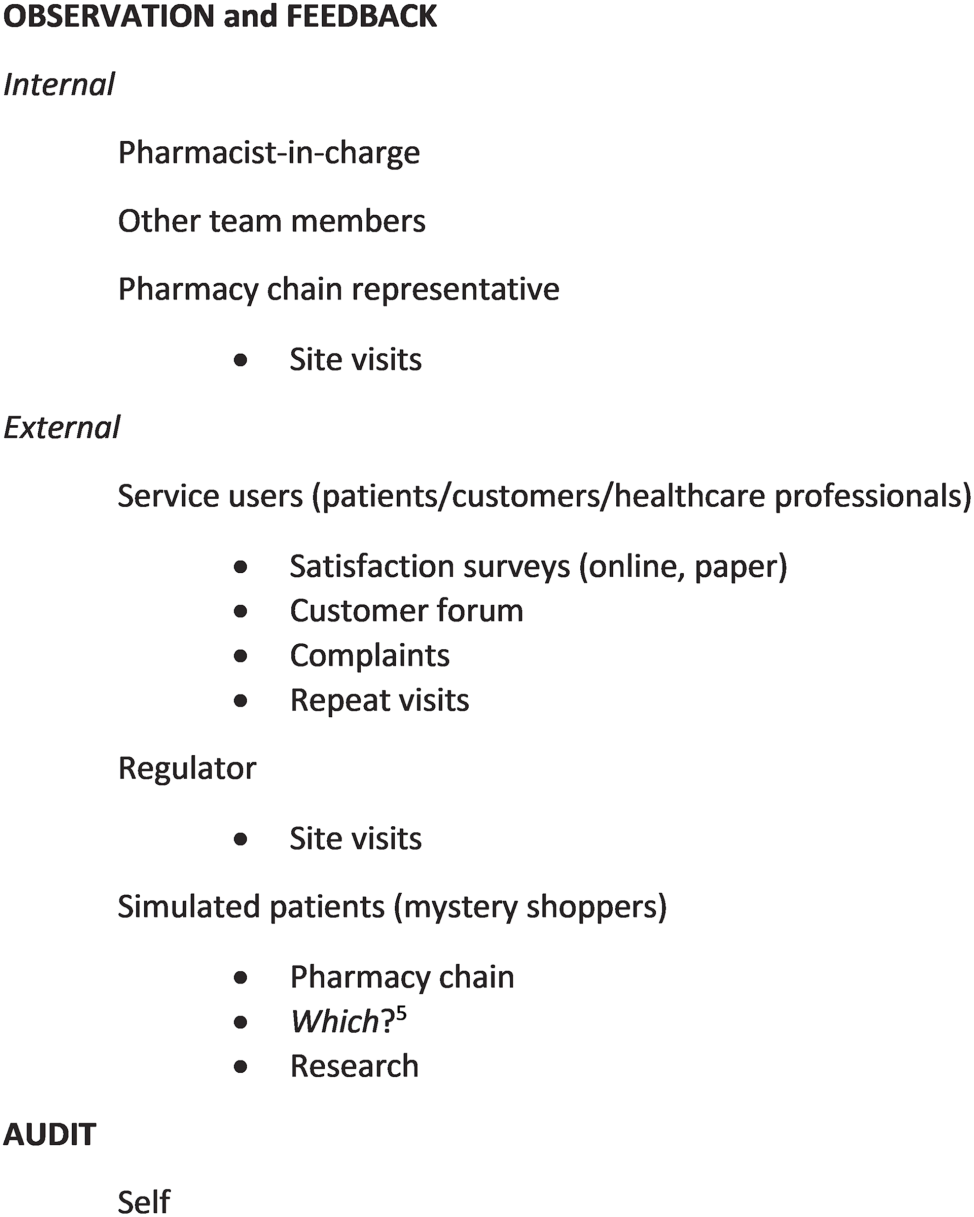
Current methods of quality measurement of community pharmacy services reported by participants.

Whilst the superintendent pharmacists (FG4) described some systematic approaches to quality measurement including the use of standards and accreditation or award schemes, overall few formal methods were reported (Fig 1) and included feedback, observation and “*other*”.

Feedback on performance from different sources and in different formats was often mentioned as a method of assessing quality. For example, negative feedback (complaints) or adverse events (which tended to be cited more frequently than positive feedback) was discussed by several participants as the impetus for assessing quality or practice:

*“Having experience as a student in various different pharmacies*, *I’ve never noticed any .particular method of the pharmacist or whatever, assessing quality*. *The only time quality of a consultation or whatever comes into play is when you get a [sic] negative feedback, there’s never positive encouragement of your staff to deliver the quality of care until something negative is said”*

*(FG1)*.

It was suggested that pharmacists are uncomfortable with reflecting and discussing encounters that have not gone well:

*“So you can train and coach and share knowledge and give real examples and go back when things haven’t gone as well*. *That’s partly I would say that’s the role of the pharmacy [sic] (pharmacist) how could that interaction have happened in a different way? People, by nature, don’t like conflict though…and in my experience, pharmacy teams especially don’t like conflict in the environment that they work in”*

*(FG4)*.

There was also discussion of learning from positive events e.g. other service providers’ good ideas/practice:

*“It’s all about sharing it and cascading it back out again*. *There’s nothing more powerful than people who’ve come up with something really good sharing it with their other colleagues and getting them to follow..”*

*(FG4)*.

Feedback was derived from a variety of sources: externally from service users (direct patient feedback, customer exit interviews), GPhC (General Pharmaceutical Council (the regulator for the pharmacy profession)) inspectors, mystery shoppers, or internally from pharmacy personnel (team members) or pharmacy chain representatives:

“*We’re constantly looking to see whether we get complaints or compliments*, *we do mystery shopping*, *we do audits*, *we have people doing branch visits*, *we’ve got GPhC inspections”*

(FG4).

Patient or customer satisfaction surveys were used to derive feedback. These included online or paper-based surveys or the use of prompts on till receipts to encourage customers to provide feedback. In addition, patient/customer forums and patient practice groups were identified.

Participants reported the use of direct observation, both covert and overt, as a method of quality assessment. Mystery shoppers (simulated patients) were mentioned by several participants. Pharmacist participants also described that they ‘observed’ through listening: *“it’s basically what we hear going on” (FG3)* but that tacit knowledge was also important: *“I think you do have a feeling when you’re a pharmacist and you visit”* (FG4): “*you walk into a pharmacy that looks organised and streamlined and you tend to find a better quality of service is being provided than a pharmacy where everyone is looking for owings* [medicine owed to patients to complete the supply of the full amount] *and just chaos” (FG3)*.

Other measures of quality which were mentioned by participants included repeat visits by customers which were cited as an indicator of whether good quality had been achieved with previous interactions:

“return visit is potentially an indicator of the fact that you’ve given good advice”

(FG4).

### Pharmacy and patient factors affecting quality of acute consultation management

**Fig 2:**
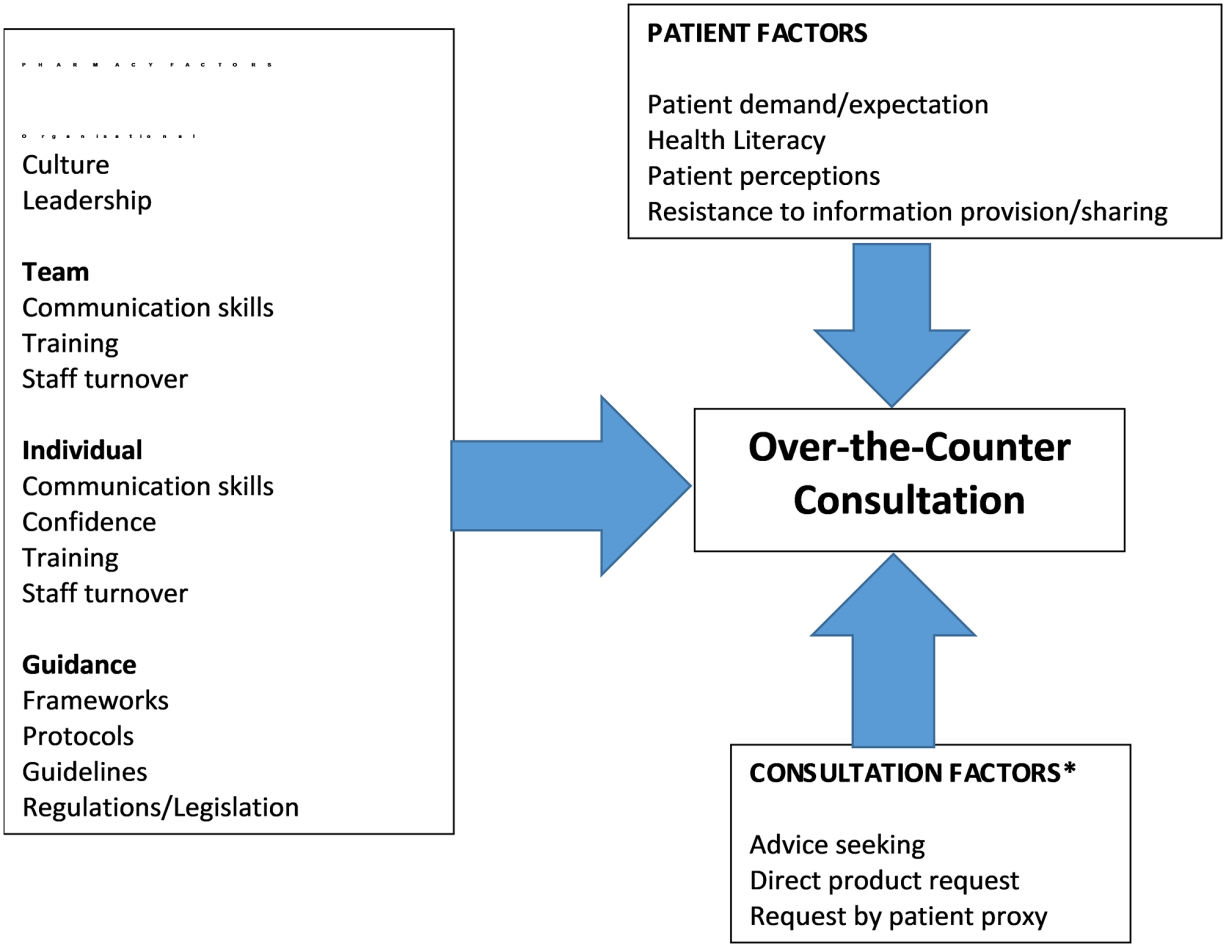
Factors perceived to influence the quality of the management acute consultations. (Watson M, Bond CM, Grimshaw JM, Johnston M. Factors predicting the guideline compliant supply (or non-supply) of non-prescription medicines in the community pharmacy setting. *Qual Saf Health*. 2006;15:53–57. [28]).

Participants identified a wide spectrum of influences on the quality of the management of acute consultations (Fig 2). These included individual, team and organisational factors, as well as patient and consultation factors. Individual characteristics associated with providing high quality acute consultations included: good communication skills (information gathering/provision); the ability to engage with patients/customers; to listen and to explain the range of treatment options; acting within one’s competence; having confidence to treat and/or to refuse to sell/supply; and to make referrals to other healthcare providers.

In terms of team considerations, pharmacist participants emphasised the importance of having confidence that their pharmacy support staff were confident and competent and that they received appropriate training. Staff confidence was also highlighted as being important in managing patient demand and expectations (see later):

*“we often use our least trained*, *least well paid staff and then are surprised when we don’t necessarily get the outcomes that we desire or would expect”*

*(INT3)*.

*“I’ve worked in quite a few different pharmacies where the confidence and competence of the support staff varies quite a lot*. *Less confident staff tend to stay behind that safety barrier of the counter*. *Some support staff refer every consultation [to the pharmacist]”*

*(FG1)*.

However, in terms of finding strategies to address this, the difficulty of staff training, particularly using a team approach, was discussed:

*“As pharmacists we don’t have PLT [Protected Learning Time (*http://www.cpdconnect.nhs.scot/news/what-is-protected-learning-time/*)]*, *we don’t have time often that we can devote to continuing our own training and staff training*.*”*

(FG1)

*“One of the key elements of quality improvement is getting the whole team involved in a service together ….but the chances of getting the whole team together* .. *is virtually zero”*

*(Int4)*.

The issue of good continuity of staff was also identified as being associated with better quality because it was believed to be important for developing trust and rapport between patients and pharmacy personnel:

*“It depends on how long the counter assistant’s been there*. *For example, where I am, she’s full-time, she knows the local community, she’s the first port of call for people when they walk in so that trust and rapport is already there. …. if you’re getting changing members of staff it can be hard to maintain any sense of quality in the conversations until they’re fully trained I think”*

(FG3).

Conversely, lack of continuity of staff and consistency regarding their approach/team-working were highlighted as factors which could negatively affect the quality of service delivery and patient care:

*“it is ..the responsibility of the pharmacist to go in there and become part of the team immediately*, .. *something to say ‘I’m in it, I’m with you, you can ask* ..*I’m approachable’ … you hear anecdotally* ..*some people [pharmacists] just come in and put a laptop on, like how is a counter assistant going to feel comfortable going over to someone and feeling like they’re interrupting them to say ‘oh can I just check this sale with you’”*

*(FG3)*.

In terms of organisational factors, participants suggested a lack of quality improvement within the existing culture of community pharmacy services. Furthermore, it was suggested that the community pharmacy sector might not be conducive to quality improvement:

*“I don’t think quality improvement* ..*has caught the imagination of community pharmacists*. *We are trained around accuracy and exactness and preciseness and they aren’t really things that lend themselves well to quality improvement”*

*(Int4)*.

Several reasons were suggested as contributing to the lack of quality improvement culture in this setting. These included lack of personal empowerment and autonomy of staff. This manifested itself in different forms including professional and commercial regulations and policies which were identified as limiting autonomy:

*“it requires a large degree of personal empowerment and management willingness to let people experiment* ..*and I think that’s often not there in community pharmacy…… I think by and large, community pharmacists feel quite helpless to do anything outside of the rigid management structure that they work within”*

*(Int4)*.

The physical environment of pharmacies was also identified as a potential barrier to delivering appropriate care:

*“there are so many barriers in a community pharmacy to asking questions*. *The fact that the counter is there; the counter is a barrier itself”*

(FG1)

Lack of public awareness regarding the reasoning behind pharmacy personnel’s attempts to elicit information during consultations led to participants suggesting that public perception was that *“it just becomes us being the police”* (FG3). Participants perceived patient resistance to, or lack of awareness of, the need for pharmacy personnel to elicit information as part of the consultation:

“*I think there’s still public perception of the pharmacist is a shopkeeper and medicines as a commodity*…*and that’s the bit where we get into this conflict about quality versus supply”*

(FG2)

“it’s almost ‘why are you asking me when I’ve seen it advertised on TV last night and it says it’s going to fix all my problems?’”

(*FG3*).

Participants also highlighted the challenge of managing patient demand arising from incomplete or incorrect knowledge:

*“patients who come in have a different knowledge base, whether it’s the right knowledge base or not* .. *People have immediate access to information, they could stand in front of you with their phone and access some information that could refute what you’re saying*. *So that’s a challenge”*

*(FG4)*.

Participants also emphasised the challenge of achieving person-centred acute consultations due to having to juggle competing demands i.e. the patient versus regulations and incomplete information. It was also suggested that current practice emphasised the provision of information by pharmacy personnel rather than checking patients’ understanding:

*“*..*the patient’s expectations maybe completely unrealistic in that they may just want to be given what they want and we have a professional and a legal responsibility to manage that situation.”*

*(FG4)*.

“We’re too focused on giving them the information and not making sure that they’re actually going away understanding that information”

(FG1).

## Discussion

This study explored the views of a range of UK service providers about quality and quality improvement in the community pharmacy setting. Throughout this study, there emerged a strong sense of pharmacists juggling and balancing competing demands and influences. These were not limited solely to the challenge of having the dual role of health service provider and commercial entity, but also providing person-centred care whilst complying with legal and clinical guidance as well as the need for better standardisation/reduced variation in practice.

### What does quality mean to community pharmacy service providers?

We explored the concept of quality (definition, measurement and improvement) in relation to community pharmacy services in general and more specifically the management of acute consultations. Whilst participants’ perceptions of quality were multi-dimensional and interlinked, reflecting the findings of Halsall et al[13], of the Institute of Medicine’s (IOM’s) six dimensions of quality[14], those which emerged strongly in the narratives of participants in this study were *safety* and *person-centred care*. Quality improvement has been defined as “*a systematic approach that uses specific techniques to improve quality*.*”*[15] Our results suggest a lack of systematic approach to the definition and measurement of the quality of community pharmacy services and a continued dominance of focus on volume-based activity or productivity.

### What quality measures are used in community pharmacies?

A wide range of actual and potential quality measures were identified none of which were used universally for any specific service. National quality indicators have been developed for community pharmacy services in a range of countries[16–18] but none have focused upon the management of acute consultations despite these being the “*shop window*” of community pharmacy. Consensus regarding what constitutes good quality pharmaceutical services has been explored in different settings[19]. In April 2017, eight quality indicators were introduced by the Department of Health for all community pharmacies in England[20] with points and reimbursement aligned to each indicator. Several indicators reflect the IOM’s definition e.g. safety, effectiveness, but none relate to the management of acute consultations. Patient involvement in the future development of quality indicators could assist in defining more person-centred pharmacy services and has been explored by Shiyanbola and Warholak in the US[21,22]. Shiyanbola’s study highlighted the emphasis which patients place on safety but interestingly both studies demonstrated that patients were only likely to be influenced by quality ratings if they were seeking a community pharmacy in a new area.

Adverse or negative experiences were often cited as indications of poor quality. Positive deviance[23,24] and appreciative enquiry[25], are methods of quality improvement based upon learning from positive experiences and events. A positive deviant is “an exemplary individual …or successful teams and organisations.” One superintendent pharmacist (FG4) described the use of high-performing pharmacists visiting underperforming pharmacists as one method of quality improvement used by her organisation. The use of “always events” rather than never events is another method which has been used to encompass person-centredness and quality improvement[26] which could also be explored for community pharmacy services.

### Implications for practice

There is a need to increase awareness of, and engagement with, quality and quality improvement in the community pharmacy sector. Initiatives are needed at organisational (regulatory, professional, commercial), team and individual levels to promote the development of evidence-based and stakeholder-informed measures and indicators which can be used to enhance the quality of service delivery in general and the management of acute consultations in particular.

### Strengths and limitations

This study included participants from across the UK. Our sample was diverse and included a range of personnel who held various pharmacy related roles and responsibilities. The option of participating in an interview or focus group enabled participants to choose based upon their availability and preference. Interviews and focus groups were conducted during the same time period. Pharmacists, including pre-registration pharmacy students, participated, with the latter participating in focus groups rather than interviews. Whilst pharmacists have overall responsibility for service delivery, the majority of acute consultations are managed by non-pharmacist personnel. The focus groups that were conducted with conference delegates in Scotland included participants with a wide range of experience and as such, less experienced pharmacists (including pre-registration pharmacy students) might have felt less able to participate. However, despite these concerns, five pre-registration pharmacy students participated and contributed to the discussions particularly in relation to their experience of working in different pharmacies. Three focus groups comprised participants who were attending professional conferences and as such, these individuals might have held different views from pharmacists who do not attend events of this type. However, we achieved a maximum variation sample of participants in terms of types of role i.e. employee, owner.

Data collection was undertaken by two experienced health service researchers who were also pharmacists. Whilst neither individual was a community pharmacist, they had insight into the context of community pharmacy and this may have given them credibility with the participants thus enabling the participants to speak freely and in-depth about the topics which were discussed. Conversely, the seniority of these researchers could have inhibited participants’ willingness to engage. However, the breadth and depth of topics covered suggests that participants felt empowered to participate. Data analysis was undertaken by one of these researchers as well as an experienced qualitative researcher who was not a pharmacist. This ensured a balanced approach to the analysis and interpretation of the data.

We were satisfied that our sample size was appropriate in terms of allowing us to answer our research questions relating to quality and quality improvement in the community pharmacy setting[27]. We chose to adopt an interpretive approach to the analysis rather than selecting a specific theory, to enable us to explore participants’ interpretation and sense-making of their experiences. We were confident that key recurring themes relating to quality were apparent in our data and that no new themes were emerging in the later interviews or focus groups that would necessitate further exploration with subsequent additional interviews or focus groups.

## Conclusions

This novel study has highlighted a range of topics which could be developed to enhance the quality of community pharmacy services in the UK and beyond. The challenge of achieving and balancing greater standardisation whilst delivering person-centred care needs to be addressed including engagement in co-production of quality indicators and the adoption of quality improvement techniques which build upon positive events and experience.

## Supporting information

S1 Appendix Topic guide. (DOCX)
