# Supplementary material for: Jugglers and tightrope walkers: The challenge of delivering quality community pharmacy services
Source: PLoS One. 2018 Jul 23;13(7):e0200610. doi: 10.1371/journal.pone.0200610 (PMC6056049; doi:10.1371/journal.pone.0200610)
Supplement: S1 Appendix — (DOCX) [file pone.0200610.s001.docx]

Appendix 1 Topic Guide

- What does quality mean to community pharmacists, their teams and pharmacy organisations in:
  - general
  - relation to the management of acute consultations in particular?
- How is quality and quality improvement achieved/measured in community pharmacy in:
  - general?
  - relation to the management of acute consultations in particular?
- What are your attitudes/beliefs towards/about quality and quality improvement in community pharmacy in:
  - general?
  - relation to the management of acute consultations in particular?
- How could/should quality/improvement be achieved/measured in future?
- What are your attitudes towards adopting Patient and Public Involvement methods:
  - in the development of quality guidelines
  - and more generally for community pharmacy practice?
